# Analysis of growth cone extension in standardized coordinates highlights self-organization rules during wiring of the *Drosophila* visual system

**DOI:** 10.1371/journal.pgen.1009857

**Published:** 2021-11-03

**Authors:** Weiyue Ji, Lani F. Wu, Steven J. Altschuler

**Affiliations:** 1 Biophysics Graduate Group, University of California, San Francisco, San Francisco, California, United States of America; 2 Department of Pharmaceutical Chemistry, University of California, San Francisco, San Francisco, California, United States of America; Harvard Medical School, Howard Hughes Medical Institute, UNITED STATES

## Abstract

A fascinating question in neuroscience is how ensembles of neurons, originating from different locations, extend to the proper place and by the right time to create precise circuits. Here, we investigate this question in the *Drosophila* visual system, where photoreceptors re-sort in the lamina to form the crystalline-like neural superposition circuit. The repeated nature of this circuit allowed us to establish a data-driven, standardized coordinate system for quantitative comparison of sparsely perturbed growth cones within and across specimens. Using this common frame of reference, we investigated the extension of the R3 and R4 photoreceptors, which is the only pair of symmetrically arranged photoreceptors with asymmetric target choices. Specifically, we found that extension speeds of the R3 and R4 growth cones are inherent to their cell identities. The ability to parameterize local regularity in tissue organization facilitated the characterization of ensemble cellular behaviors and dissection of mechanisms governing neural circuit formation.

## Author summary

One of the most fascinating questions in neuroscience is how complex neural circuits form. Here, we investigate self-organization principles during neuronal development in the visual circuit of fruit flies. We utilized the regularity of the tissue environment to establish a developmental coordinate system during wiring, which allowed us to uncover a role for cell identity in governing neuronal growth cone extension speed. Our study highlights the importance of velocity control in the precise formation of complex neuronal circuits, as well as the use of developmental coordinates in identifying ensemble behaviors of tissue patterning.

## Introduction

Convergence of neurons at the same time and place can be crucial for subsequent interactions, such as synaptic competition [1–3]. Yet, ways in which this is achieved, beyond the presence of guidance cues, is less well understood. The *Drosophila* visual system—in which thousands of neurons swap relative positions and identify targets with astonishing accuracy—offers a remarkable opportunity to identify cell autonomous and non-autonomous principles underlying how such kinetic patterning can occur.

**Fig 1:**
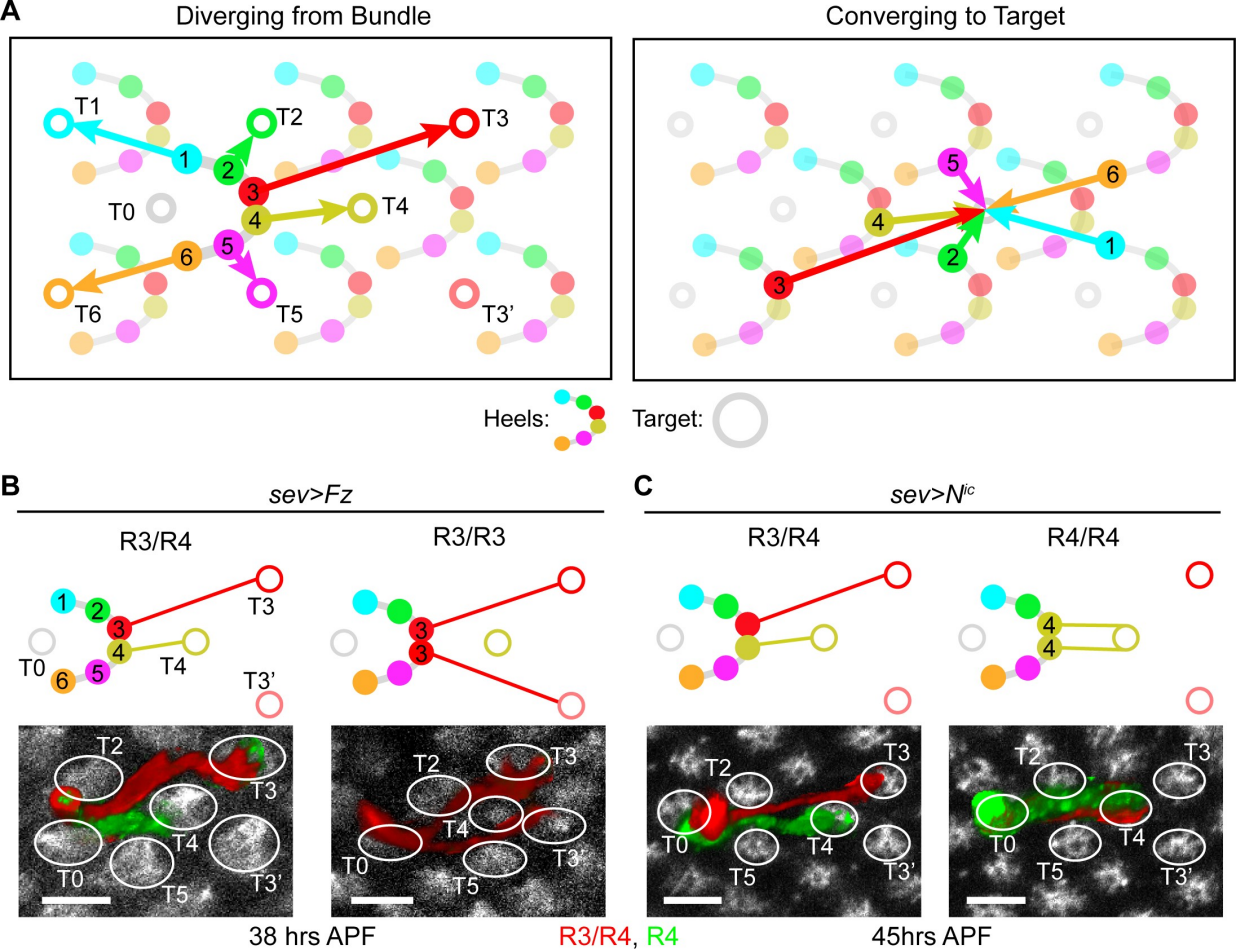
Changes in R3 or R4 cell identity lead to changes in final targeting. **(A)** Schematic of NSP wiring topology. Solid circles: Landing points of R1-R6 at the lamina (“heels”); open circles: target locations of R1-R6 growth cones. T3’: target of fate-transformed R3s; T0: target located within the bundle of interest (though targeted by R cells from other bundles in NSP wiring). R1-R6 are color coded consistently in all schematics. **(B-C)** Representative schematics and images of bundles from (B) *sev>Fz* (38 hrs APF) and (C) *sev>N*^*ic*^ (45 hrs APF) flies (see S1B and S1C Fig for more examples). Top panels: schematics of wild-type or altered wiring topology. Bottom panels: confocal images of representative bundles. Photoreceptor growth cones were segmented, pseudo-colored, and intensity scaled for visualization (Materials and Methods). Red: *sev>RFP* expression; green: *mδ0*.*5-GFP* expression; white: Fasciclin 2 (FasII) antibody staining. White ellipses: targets. Scale bar: 5 μm.

The compound eye of the fruit fly is comprised of 800 ommatidia (unit eyes), each containing 8 photoreceptors of distinct types (R1-R8). Axons of the photoreceptors extend from the eye to the optic lobe in groups of 800 “bundles” with canonical intra-bundle orientations. Six of the eight R cells (R1-R6) stop extending at the lamina plexus and wire to their postsynaptic targets. In each bundle, the R1-R6 cells diverge towards six different target positions (T1-T6); and, at each target position, R1-R6 cells converge, each cell originating from a different bundle (Fig 1A). This remarkable axonal resorting process in the lamina is referred to as neural superposition (NSP) [4–7].

An intriguing discovery is that R1-R6 each exhibit relatively consistent and cell type-specific velocity during growth cone extension [8]. This suggests that the velocity of growth cones plays a pivotal role in their synaptic partner selection. Previous studies suggested that both ommatidial orientation of the originating bundle [9] and interactions among growth cones within the same and neighboring bundles [10,11] contribute to the direction of R cell projections. However, how extension velocity of photoreceptor growth cones is controlled remains unclear.

To address this question, we developed a data-driven standardized coordinate system around each bundle built upon the lattice-like structure of the NSP circuit. This enabled the characterization of ensemble behaviors of photoreceptor cell types, despite morphological variability and stochastic differences of individual neurons and local warping of the lattice. We used this quantitative framework, together with classic fly genetics and state-of-the-art sparse labeling technique, to investigate the influence of cell identify on R3 and R4 growth cone velocity. These quantitative studies helped assess the degree to which self-organization mechanisms control the speed and direction of growth cone extension during NSP circuit formation.

## Results

### Changes in R3 or R4 cell identity lead to changes in final targeting

The early role of photoreceptor identity during development has been extensively studied. R1-R6s develop in three sequential pairs during eye development: R2/R5, then R3/R4 and last R1/R6 [12–14]. The R3/R4 pair is particularly important in breaking symmetry, including the 90° rotation of R-cell clusters in the developing eye disc and the asymmetric trapezoidal arrangement of the adult ommatidia [15,16]. Interestingly, the R3/R4 pair also breaks symmetry of the NSP wiring diagram. The target positions of R3 and R4 (“T3” and “T4”, respectively) are asymmetrical, while the target positions of the other two pairs (R1/R6, R2/R5) are symmetrical (Fig 1A). Thus, we focused our effort on understanding the role of R3/R4 identities—and their contribution to asymmetric targeting—during the NSP wiring process in the lamina.

To alter R3 and R4 cell identities, we used genetic perturbations in the planar-cell-polarity pathway. Specifically, over-expression of Frizzled (Fz) with *sevenless* (*sev*) enhancer (*sev>Fz*) generates ommatidia with two R3s [17,18], while over-expression of the intracellular domain of Notch (N^ic^) under the same enhancer (*sev>N*^*ic*^) generates ommatidia with two R4s [19,20]. To visualize the perturbed bundles, we used a membrane-bound red fluorescent protein (tdTomato) under the same enhancer. Since the lamina plexus is densely packed with photoreceptors, it is challenging to disambiguate individual photoreceptor growth cones. Thus, we induced our perturbation sparsely, utilizing an FRT-dependent GAL80 “flip-in” construct together with a heat-shock activated flippase [21,22]. Further, to differentiate between R3 and R4 cell types at an early stage of development in both the eye and lamina, we utilized the R4-specific enhancer *mδ0*.*5* fused with a membrane GFP protein (*mδ0*.*5-GFP*) [19].

We visually inspected specimens of sparsely perturbed *sev>Fz* and *sev>N*^*ic*^ flies after the completion of NSP wiring (> 36 hours after puparium formation, or hrs APF; see S1A Fig for timeline [7]). We found that changes in cell identity resulted in changes in final targeting (Figs 1B and 1C, S1B and S1C). For bundles with two R4 photoreceptors, both the normal and fate-transformed R4s target the same canonical R4 target, T4. For bundles with two R3 photoreceptors, the normal R3 targets the canonical R3 target, T3, while the fate-transformed R3 targets T3’, a new target position that is mirror-symmetric to T3, instead of the original T3. (See Table 1 for phenotype penetrance.) This result raised the hypothesis that cell identity and the position of cells within the bundle could influence the targeting of R3 and R4 photoreceptors.

**Table 1:** Penetrance of target alteration of *sev>Fz* and *sev>N*^*ic*^.

Genetics	Age (hrs APF)	Number of bundles with altered targeting	Number of bundles with wild-type-like targeting	Total number of bundles	Penetrance (%)
*sev>Fz*	38	15	21	36	41.7
*sev>N* ^*ic*^	45	48	1	49	98.0

Images of *sev>Fz* specimens at 38 hrs APF and *sev>N*^*ic*^ specimens at 45 hrs APF were visually inspected and counted. Only bundles with two R3s (bundles with two RFP-positive R cell growth cones and no GFP-positive R cell growth cones) or bundles with two R4s (bundles with two GFP-positive R cell growth cones) are included in the counting. n = 13 biological replicates for *sev>Fz* flies and n = 5 biological replicates for *sev>N*^*ic*^ flies. We note that the targeting penetrance we observe is on par with that of previous studies in both this system [10,11] and others [23,24].

### Wild-type R3s and R4s exhibit asymmetric speeds but symmetric directions of extension

Perturbing R3 and R4 cell identity changed the final target choice. We next investigated the role of cell identity during early extension. We visually examined changes in the morphologies of R3 and R4 growth cones from early to late stage of NSP (captured every 2 hrs from 22 hrs APF to 36 hrs APF). During this time, growth cones of both R3s and R4s had one (or a small number of) long filopodia extending in the same direction as the polarized leading edge (S2 Fig). The R-cell type growth cone morphologies of fixed-tissue time series appeared visually similar across specimens and were consistent with published live-imaging data [8].

**Fig 2:**
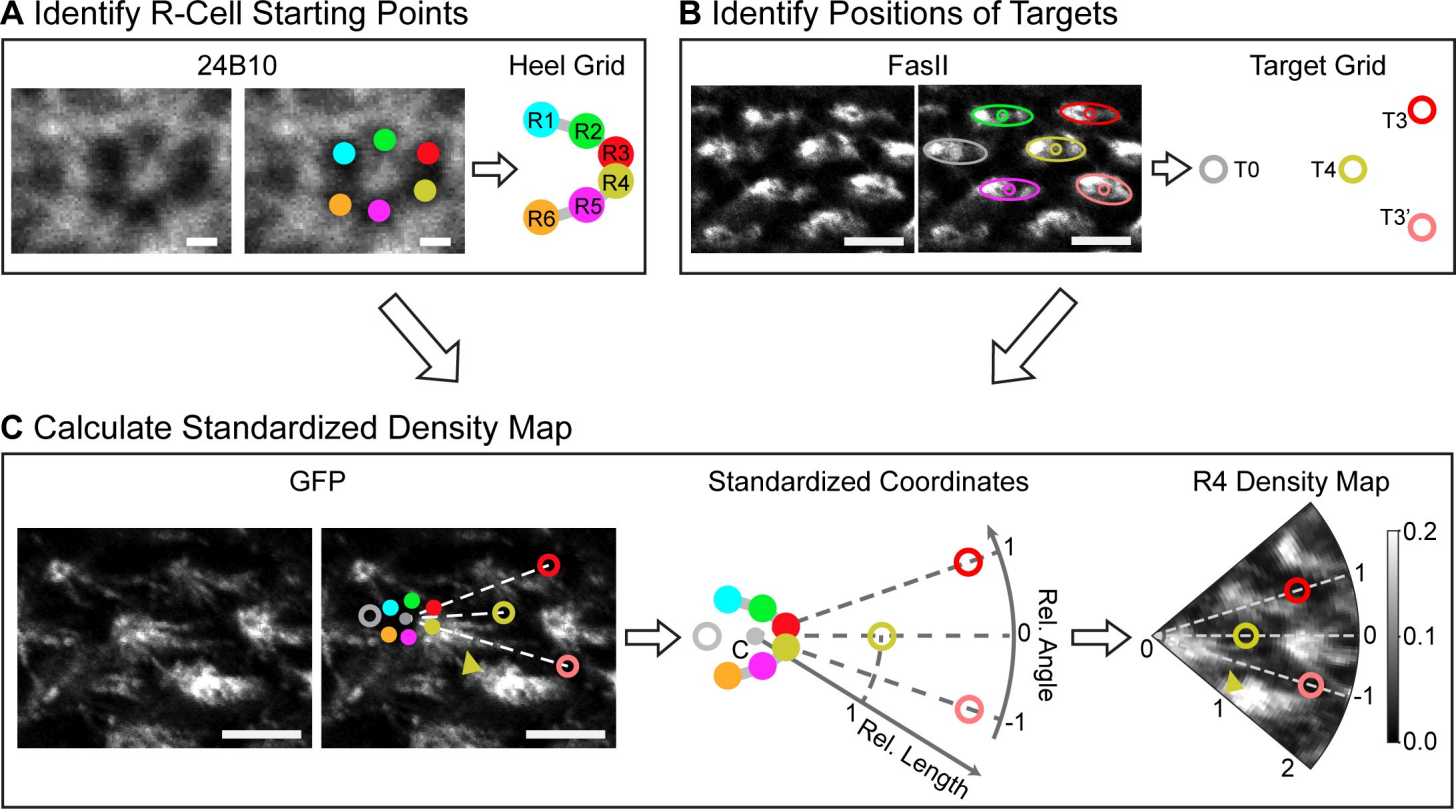
Establishment of standardized coordinates for comparison of growth cone extension. Overview of the image quantification process. **(A)** The heel grid (i.e., R-cell extension starting points) is identified (via 24B10 antibody labeling R-cell membranes; Materials and Methods). **(B)** The target grid is identified (via FasII antibody labeling L-cell membranes; Materials and Methods). **(C)** In each region, (left) the annotation (from A-B) is used (middle) to produce standardized coordinates that are used (right) to transform confocal images into standardized density maps. This transformation allows relative lengths (radial coordinate) and angles (angular coordinate) of extending growth cones to be compared across regions. Images: confocal images of a sample bundle region from one wild-type fly at 26 hrs APF. Images were cropped, re-oriented and intensity scaled for visualization (Materials and Methods). Arrowheads highlight growth cones of interest. Scale bars: 5 μm for FasII and GFP images; 1 μm for 24B10 image.

Searching for general rules governing wiring can require observing and analyzing large numbers of clearly distinguishable neurons, which may be challenging using intravital imaging. Here, we chose to quantify sparsely labeled, fixed-tissue specimens, which provided relatively large numbers of growth cones across developmental time points. However, this required development of new analytical tools, as comparative analysis of growth cones within and across tissue specimens is confounded by variations in image orientation, local warping of the lamina plexus, and inherent cell-cell variability. We developed a “standardized” coordinate system that utilizes the regularity of NSP circuit to align local bundle configurations (Fig 2; Materials and Methods). In neural superposition, there are two distinct grid-like structures: one “heel grid”, formed by the landing positions of the photoreceptors in the lamina, and one “target grid”, formed by the dendrites of their targets, the lamina monopolar cells (Fig 1A). The alignment of these two grids provides the local regularity needed to define the standardized coordinates.

For each bundle, we identified the starting positions of all R-cells (Heel grid, Fig 2A) and their putative targets (Target grid, Fig 2B). We also identified a center point “C”, which lies at the intersection of the line connecting R3 and T3 and the line connecting R4 and T3’. We then extrapolated polar coordinates by normalizing (Fig 2C): 1) length, so that |C-T4| = 1 (7.1 ± 1.5 μm before normalization); and 2) angle, so that ∡(T3,C,T4) = ∡(T4,C,T3’) = 1 (∡(T3,C,T4) = 14.8° ± 4.0°, ∡(T4,C,T3’) = 13.3° ± 3.8° before normalization), and that T3 and T3’ were placed at angles +1 and -1, respectively. The standardized coordinates, defined by the center points and normalization for each local region, allowed us to register bundles within and across specimens (S3 and S4 Figs) and identify ensemble behaviors of R3 and R4 cell types. This approach is designed to characterize relative, rather than absolute, changes in length or angle of extending growth cones with respect to the normalized and invariant target grid.

**Fig 3:**
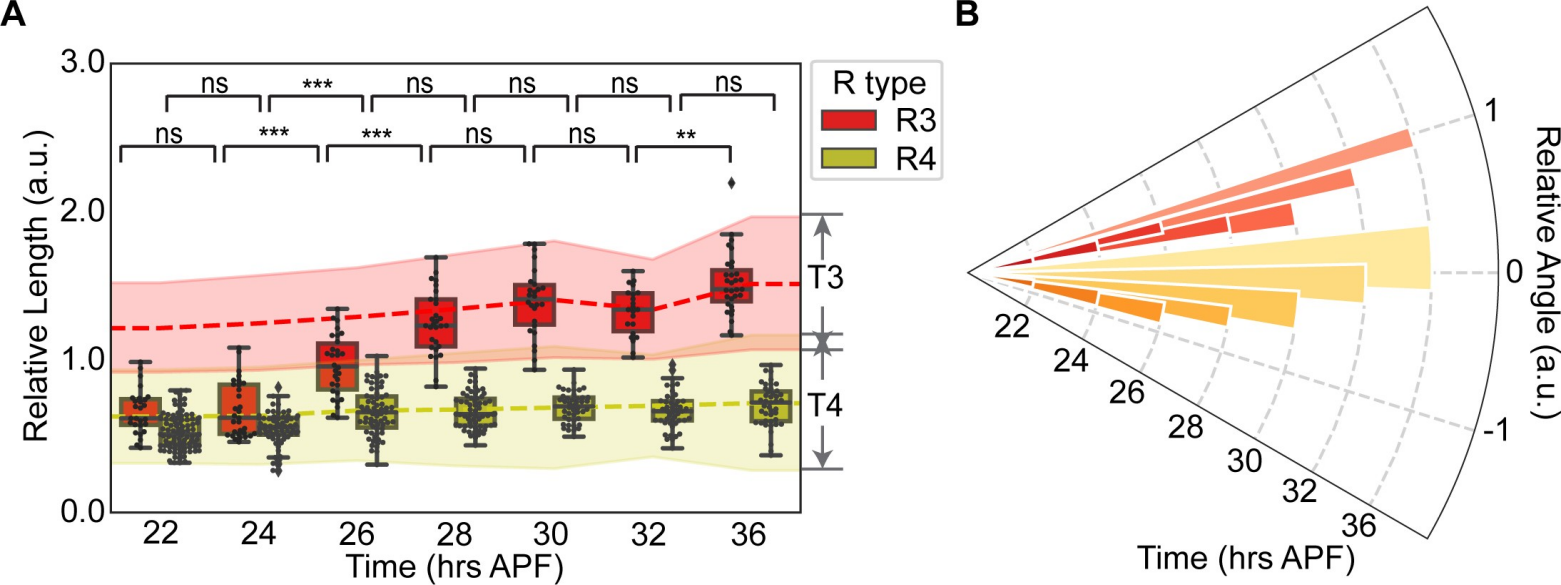
Wild-type R3s and R4s exhibit asymmetric speeds but symmetric directions of extension. **(A)** Changes in relative lengths of wild-type R3 and R4 growth cones over time. Red and yellow represent R3s and R4s, respectively. Dashed lines: mean of target centers. Error bars of the dashed lines: mean of the upper and lower boundaries of targets. Significance: calculated using a two-sided Mann-Whitney test with p values adjusted by Holm-Bonferroni method. ns: p ≥ 0.05, *: 0.01 ≤ p < 0.05, **: 0.001 ≤ p < 0.01, ***: p < 0.001. Sample sizes (number of bundles) of each time point: R3 growth cones (n = 27, 31, 30, 29, 26, 22, 31); R4 growth cones (n = 93, 67, 67, 66, 44, 46, 43). n ≥ 2 biological replicates for each time point. **(B)** Changes in relative angles of wild-type R3 and R4 growth cones over time. Red and yellow bars represent R3s and R4s, respectively. Radial coordinate indicates time, angular coordinate indicates mean relative angle of R3 or R4 growth cones at the given time point, and width of bars indicates standard deviation of angle values at the given time point. Sample sizes as in (A). See S2 Fig for representative images of each time point. See S1 Table for p-values and S1 Data for data used to generate this figure.

For each R3 or R4 growth cone, we estimated its angle and speed towards their putative targets. We chose to define the “front” of the growth cone by the average fluorescence intensity at the leading edge (Materials and Methods); this measurement of leading-edge filopodia provided a fiducial for approximating growth cone extension. Based on ensemble measurements, R3 and R4 arrive at their target regions by 28 hrs APF (Fig 3A). However, R3 travels considerably further and therefore must have higher extension speed (in agreement with previous findings [8]). Further, R3 and R4 initially have symmetric extension angles until they reach their targets at 28 hrs APF (this symmetry breaks after 28 hrs APF when the leading edges adhere to their asymmetrically positioned targets; Fig 3B).

### Extension speed is instrumental for asymmetrical targeting

**Fig 4:**
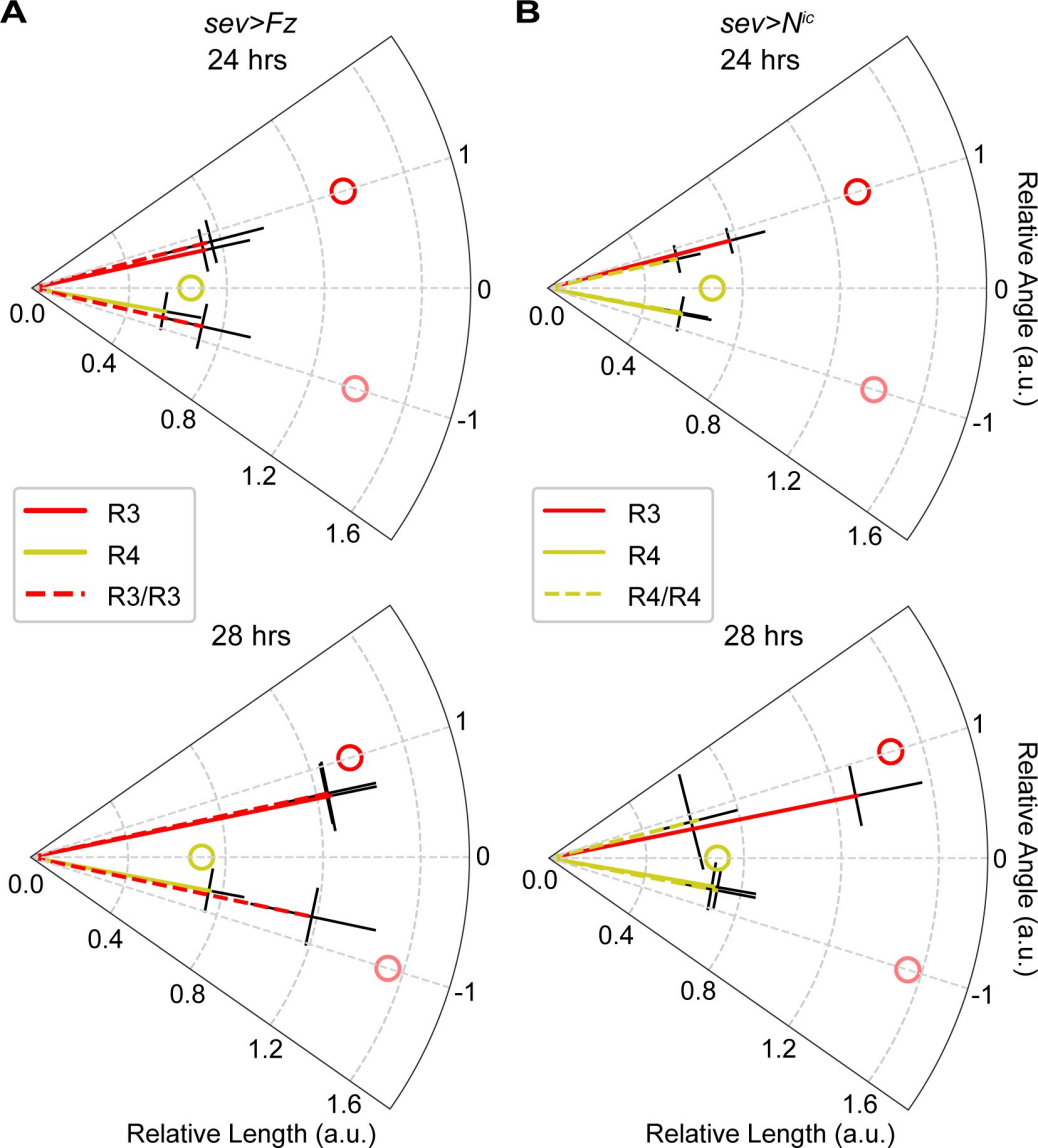
Extension speed is instrumental for asymmetrical targeting. Polar plots of relative lengths and angles for wild-type-like (R3/R4) and fate-transformed (R3/R3 or R4/R4) bundles under **(A)** *sev>Fz* and **(B)** *sev>N*^*ic*^ conditions at 24 and 28 hrs APF. See S5 Fig for representative images of each condition. Radial coordinate: relative length; angular coordinate: relative angle; error bars: standard deviations for length or angle. Solid lines: control bundles; dashed lines: fate-transformed bundles. Open circles: mean of target centers. Sample numbers for growth cones at wild-type R3 position, wild-type R4 position, perturbed R3 position, and perturbed R4 position, respectively: *sev>Fz* at 24 hrs (n = 32, 47, 13, 13) or 28 hrs (n = 52, 143, 22, 22); *sev>N*^*ic*^ at 24 hrs (n = 23, 46, 10, 10) or 28 hrs (n = 39, 113, 29, 29). n ≥ 3 biological replicates for each genetics and time point. See S1 Table for p-values and S1 Data for data used to generate this figure.

Based on these observations in wild-type, we hypothesized that extension speed plays a key role in the asymmetrical targeting of R3/R4 pairs. To test this hypothesis, we examined the behavior of sparsely perturbed mutant (*sev>Fz* and *sev>N*^*ic*^) fly specimens that have both wild-type-like and fate-transformed bundles at 24 or 28 hrs APF, corresponding to early or late stages of their extension (Figs 4 and S5).

We compared the ensemble behaviors of fate-transformed to wild-type bundles. The fate-transformed bundles contain either two R3s or two R4s (one of which is fate transformed and the other wild-type). We observed that the extension speed and angle of the wild-type cells in the fate-transformed bundles is similar to the R cells of the same fate in the wild-type-like bundles. More importantly, we discovered that within the fate-transformed bundles, the two R3s or two R4s extend at similar speeds and symmetric angles (Fig 4). Thus, we concluded that cell fate is instrumental in determining extension speed of photoreceptor growth cones, and the asymmetrical speeds of R3 vs R4 ultimately result in their asymmetrical targeting.

## Discussion

Individual cells within developing tissues need to make decisions about their movement velocities in order to arrive at a final collective pattern [25,26]. A fascinating instance of collective cell migration is the patterning of neuronal circuits. How is velocity—defined by both the direction and speed of extending growth cones—controlled during neuronal development to achieve proper circuit formation? Here, we investigate this question in the context of NSP circuit, in which ~4800 neurons (= 800 bundles x 6 R-cell types) swap relative positions and identify their targets with astonishing accuracy [4,11,27–29].

To compare growth cone velocity across populations of neurons, we developed a standardized coordinate system for describing growth cone morphology. This coordinate system was essential in overcoming bundle-to-bundle and fly-to-fly heterogeneity, and similar approaches could be adapted to quantify and compare the dynamics of neurons in other developmental systems with other wiring geometries. We found that cell identities for R3 and R4 neurons determine their speeds but not directions of extension. As a consequence, while R3 and R4 start with symmetric extension directions, their differences in speed lead to extension length differences by the final time of wiring and subsequently to asymmetric target choices. These observations highlight a crucial role for cell-autonomous mechanisms in controlling the dynamics of neuronal extension and, ultimately, the spatial-temporal coincidence of presynaptic and postsynaptic neurons.

In this study, we focused on the mechanism that regulates the extension speed of photoreceptor growth cones. What self-organizing mechanism might influence their extension direction? An appealing hypothesis, suggested by previous work [10,11], is through repulsion of neighboring growth cones within the same bundle. Our quantitative data suggests that a simple repulsion model—where each R cell contributes equally—is not sufficient to explain the measured extension direction; rather, a model is supported in which R2/5 contributes much more strongly than R3/4 (S6 Fig). Such differential repulsion could arise through different strengths of expressed repulsive cues among neighboring cells. For example, Flamingo, a seven-pass transmembrane cadherin capable of inducing repulsion in both axons and dendrites [30,31], is differentially expressed amongst the different R-cell types, with R2/R5 having ~3x the expression levels of R3/R4 [11]. Our standardized coordinate system provides a quantitative framework for future investigations into mechanisms that influence the initial extension directions of neuronal wiring.

Our study also provides a functional readout and framework for future investigations into mechanisms of how cell identity controls growth cone dynamics. Regulation of cytoskeletal dynamics is a clear possibility. Studies in cultured mammalian neurons have identified key components of the cytoskeleton that influence axon outgrowth and how these components can be regulated by signaling molecules [32,33]. For example, the cell-surface receptor Notch has been shown to regulate the speed of neurite outgrowth [34–36] via controlling the stability of microtubules [37] and the expression levels of signaling proteins [38]. Since Notch is both key in R3/R4 fate determination and has the potential to influence extension speed, these two properties may be intertwined, and further investigation is needed to fully understand the role of Notch in NSP wiring. Additionally, while we focused on extension velocity, our findings do not exclude other factors that may also contribute to growth cone wiring that are also changed when cell identity is transformed. Understanding how cell identities translate to differences in molecular profiles and finally to changes in the cytoskeletal networks that control growth cone morphology and filopodial dynamics [39], will provide valuable insight into how extension velocity can be controlled during neuronal circuit development *in vivo*.

Coordinated arrival of neurons is thought to be crucial in ensuring point-to-point connectivity of multiple mammalian complex circuits, including the neuromuscular map, ocular dominance columns in the visual cortex, and Purkinje cell wiring in the cerebellum [1,40–42]. Further, changes in the kinetics of developing neurons have been observed in multiple contexts, such as midline crossing of retinal axons [43–45] and commissural neurons [46], and the migration of Mauther cells across successive motor neurons [47]. Our findings in the *Drosophila* neural superposition circuit provide a case study that connects neuronal kinetics with circuit formation, highlighting the importance of velocity control in ensuring the proper convergence of presynaptic neurons, and subsequently, the precise formation of a complex neuronal circuit.

## Materials and methods

### Fly stocks and handling

Fly stocks were constructed and maintained at 25°C using standard protocols.

The following fly lines were used in this study: tubP>stop>GAL80 (II), tubP>stop>GAL80 (III) and hs-FLP122 (X) (gifts from T. Clandinin, Stanford University), mδ0.5-CD4::GFP (III) (gift from P.R. Hiesinger, Freie Universität Berlin), Uas-N^ic^ (II) (gift from B.A. Hassan, Paris Brain Institute); UAS-CD4::tdTomato (II) (gift from L.Y. Jan and Y.N. Jan, UCSF), UAS-fz1-1 (III) (Bloomington Drosophila Stock Center # 41791) and sevEP-GAL4.B (II) (Bloomington Drosophila Stock Center # 5793).

For experiments with pupae specimens, pupae with the correct genotype were collected at 0 hrs APF and aged at 28°C to increase the penetration of genetic perturbations[48]. Both male and female were used for all experiments.

### Heat shock clone induction

Larvae with the correct genotype were heat shocked for 8–15 mins at 37°C 2 to 4 days after egg laying (AEL).

### Immunohistochemistry and imaging of pupae brains

#### Dissection and staining

Pupal brains were dissected at the appropriate developmental stages in PBS (Phosphate Buffered Saline) and fixed with 3.7% formaldehyde-PBS for 30 mins. Fixed brains were washed three times in PBT (PBS with 0.4% Triton X-100) at room temperature and then blocked with PBT-BSA (3% Bovine Serum Albumin in PBT) for 1 hour. Two rounds of antibody staining were then performed. In each round, brains were incubated with cocktails of primary antibodies at 4°C overnight, rinsed in PBT, then incubated with cocktails of secondary antibodies at 4°C overnight or for 2 hrs at room temperature, then rinsed again in PBT.

#### Antibodies

Primary antibody used for the first round is: mouse anti-Fasciclin II (DSHB, 1D4), 1:20. Conjugated secondary antibody used for the first round is: Goat anti-mouse Alexa-405 (A31553, Thermo Fisher Scientific), 1:100. Primary antibodies used for the second round are: mouse anti-Chaoptin (DSHB, 24B10), 1:20; Chicken anti-GFP (abcam, ab13970), 1:400; Rabbit anti-RFP (Rockland, 600-401-379), 1:400. Conjugated secondary antibodies used for the second round are: Donkey anti-mouse Alexa-647 (A31571, Thermo Fisher Scientific), 1:100; Goat anti-chicken Alexa-488 (A32931, Thermo Fisher Scientific), 3:500; Donkey anti-rabbit Alexa-568 (A10042, Thermo Fisher Scientific), 1:500.

#### Mounting and imaging

Specimens were mounted in VECTASHIELD Antifade Mounting Medium. Specimens were mounted so that the eye imaginal disk is on the top next to the cover slip, and the optic lobe is on the bottom. Images were obtained on a Nikon A1R-Si inverted confocal microscope with 4 line laser unit (405/488/561/640) and with a 60X oil objective. Z stacks were acquired with a step size of 0.125 μm between optical sections.

### Image processing for visual inspection and figure generation

Images were transformed from ND2 format to TIF format and background subtracted in batch using custom ImageJ macro script on a HPC cluster (code available on GitHub: https://github.com/AltschulerWu-Lab/nsp_extension).

To calculate the penetration of *sev>Fz* and *sev>N*^*ic*^ perturbation (Table 1), images of *sev>Fz* lamina specimens at 38 hrs APF and *sev>N*^*ic*^ lamina specimens at 45 hrs APF were visually inspected using Fiji (http://fiji.sc/) and targets of bundles with two R3s (bundles with two RFP-positive R cell growth cones and no GFP-positive R cell growth cones at R3 and R4 positions) or two R4s (bundles with two GFP-positive R cell growth cones at R3 and R4 positions) were counted. Only bundles with R cell growth cones that can be easily traced from origin to target were included in the counting. *sev>Fz* and *sev>N*^*ic*^ perturbation were scored at different developmental time points due to the decay of quality of GFP signal in *sev>Fz* specimens over time.

To generate representative images of bundle targeting phenotypes (Figs 1B and 1C, S1B and S1C), we annotated cropped confocal images of *sev>Fz* specimens at 38 hrs APF and *sev>N*^*ic*^ specimens at 45 hrs APF in Amira 2020.1 (FEI Visualization Sciences Group). For each cropped image stack, growth cones from one bundle were manually segmented in both GFP and RFP channels. Segmented growth cones were then rendered with the appropriate color (red for RFP and green for GFP) in volume, and the unsegmented FasII channel was overlaid as an ortho-slice in grayscale. TIF files of the results were then exported from Amira and further annotated using Adobe Illustrator. To show bundles in consistent orientations, some images were rotated and/or flipped. Images were also cropped to highlight the representative bundle.

To generate representative images of bundle extension phenotypes (Figs 2, S2 and S5), images of sample bundles from 22 to 36 hrs APF were inspected and adjusted using Fiji (http://fiji.sc/). Brightness and contrast of individual channels were adjusted separately, and only one z-stack was selected for visualization. TIF files of individual channels were exported from Fiji and further annotated using Adobe Illustrator. To show bundles in consistent orientations, some images were rotated and/or flipped. Images were also cropped to highlight the representative bundle.

### Image quantification using standardized coordinates

#### Pre-preprocessing

Images were transformed from ND2 format to TIF format and background subtracted in batch using custom ImageJ macro script on a HPC cluster (code available on GitHub: https://github.com/AltschulerWu-Lab/nsp_extension).

#### Annotation

Images were visually inspected, cropped and annotated using Fiji (http://fiji.sc/). Due to the variation in mounting and sparseness of labeling, only images of the lamina plexus with large intact regions were further analyzed. Images were cropped (in all directions) to keep the part of the lamina plexus that had sparse-enough labeling of growth cones. These cropped images were then used to manually annotate the position of growth cone heels (starting positions) and targets. Heels of growth cones were annotated based on the 24B10 channel using the “Multi-point tool”, while targets were annotated based on the FasII channel using the “Elliptical selections” tool. Multiple z-slices were used to annotate target ellipses to better represent the boundaries of FasII staining. X, Y positions of heels and major, minor axis lengths and the major axis angle of the target ellipse were exported to a csv file for later quantification. Mapping of each bundle number to its corresponding target numbers was also noted in another csv file. Bundles with rotational defects were not included in the annotation.

#### Quantification

*Standardized coordinate system*. We used custom Python scripts (code available on GitHub: https://github.com/AltschulerWu-Lab/nsp_extension) to resample GFP and RFP images of each bundle to obtain representative density maps according to a standardized coordinate. Our standardized coordinate system (see Fig 2) resembles a polar coordinate system. The center of the coordinate system (C = (0,0)) is defined as the intersection of the lines connecting R3 and T3 and R4 and T3’. The polar coordinate is normalized so that: 1) the radius |C-T4| = 1 (A.U.) and 2) ∡(T3,C,T4) = ∡(T4,C,T3’) = 1, and that the targets T3 and T3’ are placed at angles +1 and -1 (A.U.), respectively. The centers of the target ellipses were used as reference points for the standardized coordinate system.

*Density map of image slice*. We converted image data to a density map in our standardized coordinates in two steps. First, we created a coordinate grid (the radius ranged from 0 to 3.8 with 0.05 intervals; the angles ranged from -3 to 3 with 0.05 intervals). Second, we used the N-dimensional piecewise linear interpolation function within the Python numpy package (v1.16.4)[49] to create a map from Cartesian to polar coordinates; this allowed us to convert GFP and RFP images into density maps in the new coordinate system.

*Density map of bundle*. Density maps for each bundle were computed using the mean density map across z-stacks containing 41 slices, which were centered around the z-slice showing the longest growth cone (typically R3 and/or R4). We manually annotated the length and angle of R3 or R4 growth cones of a given bundle according to the RFP or GFP density map, respectively. Length and angle of growth cones were annotated based on the long filopodia in the front of the growth cone. Growth cones located in regions where GFP or RFP signals were too dense to distinguish the front were excluded from the annotation. If growth cones exhibit split morphology (i.e., two or more major long filopodia in the front), angles were calculated by the mean of these filopodia. Growth cones were labeled as R3 or R4 based on the absence or presence (respectively) of strong GFP signals.

### Comparison of standardized coordinates across specimens and time points

Coordinates of heel and target positions of analyzed bundles measured from the original images can be obtained during the quantification process described above. To align them for visualization, coordinates are centered around the intersection of the lines connecting R3 and T3 and R4 and T3’ (i.e. C, the center of the standardized coordinate system), and rotated so that C-T4 align with the x-axis. Coordinates of the R3/R4 heel positions and T3/T4/T3’ target positions after our standardized coordinate transformation can also be obtained during the quantification process described above. Those coordinates are plotted as polar plots for visualization.

### Simulation of repulsion model

Heel and target positions were mapped to the standardized coordinate system. Simulated extension angles ($\vec{v_{p}}$) were calculated based on weighted vector sum of two vectors ($\vec{v_{1}},\vec{v_{2}}$). For extension angles of R3, the repulsion vectors: $\vec{v_{1}}$ was taken to be the unit vector from the R2 to R3 heels; and $\vec{v_{2}}$ was taken to be the unit vector from the R4 to R3 heels. For extension angles of R4, the repulsion vectors: $\vec{v_{1}}$ was taken to be the unit vector from the R5 to R4 heels; and $\vec{v_{2}}$ was taken to be the unit vector from the R3 to R4 heels. The weight of each vector represented the strength of its repulsive force. For simple repulsion from neighboring heels, $\vec{v_{p}}=0.5\;\vec{v_{1}}+0.5\;\vec{v_{2}}$. To estimate unequal influence of neighboring growth cone heels, linear regression was performed on $\vec{v_{p}}=\alpha \;\vec{v_{1}}+\beta \;\vec{v_{2}}$ using pooled data from wild-type measurements between 22 to 26 hrs APF. Only data from bundles that were relatively symmetric in shape ($\frac{\left|∡(T3,C,T4)-∡(T3\prime ,C,T4)\right|}{max\{∡(T3,C,T4),∡(T3^{\prime },C,T4)\}}\leq 0.5$) were included in the regression analysis. R3 and R4 angles were fitted independently. Regression analysis was implemented in Python using the scikit-learn package (v0.23.2)[50].

### Statistical analysis

Sample sizes for each experiment are provided in the figure legends. Statistics were computed in Python using the scipy (v1.2.1)[51] and scikit-posthocs (v0.6.4)[52] packages. A two-sided Mann-Whitney U test was applied when there were only two groups of data being compared. When there were more than two groups, we applied a Kruskal–Wallis H test followed by a post-hoc two-sided Mann-Whitney test with p values adjusted by Holm-Bonferroni method. The error bars displayed in all figures represent standard deviation.

## Supporting information

S1 Fig Timeline of neural superposition and final targeting of *sev>Fz* and *sev>N^ic^* flies. **(A)** Schematic for timing of NSP wiring. APF: “After Puparium Formation”. **(B-C)** Schematics (top panels) and confocal images (bottom panels) of bundles from three (B) *sev>Fz* (38 hrs APF) and (C) *sev>N*^*ic*^ (45 hrs APF) specimens. Top panels: schematics of wild-type or altered wiring topology. Solid or open circles: starting points (‘heels”) or targets (respectively); colors coordinated between R cells and targets. T3’: target of fate-transformed R3s; T0: target located within the bundle of interest (though targeted by R cells from other bundles in NSP wiring). Bottom panels: confocal images of representative bundles. Photoreceptor growth cones are segmented and pseudo-colored (Materials and Methods) and intensity scaled for visualization. Red: *sev>RFP* expression; green: *mδ0*.*5-GFP* expression; white: Fasciclin 2 (FasII) antibody staining. White ellipses: targets. Scale bar: 5 μm.
(TIF)

S2 Fig Extension phenotype of wild-type flies over time. Representative images of bundles from wild-type flies from 22 to 36 hrs APF. Left: Raw images of representative bundles. From left to right: FasII channel labeling the target cells; 24B10 channel labeling membrane of all R cells; GFP channel labeling membrane of R4 cells; RFP channel labeling membrane of R3 or R4 cells. Right: Density maps of GFP (R4 cells) and RFP channel (R3 or R4 cells) after coordinate transformation. For visualization, intensity is scaled differently for each channel and for each sample. R-cells and targets are indicated and colored as in S1B Fig; white circles: T0; gray circles: other targets. Yellow arrowheads: R4 growth cones; red arrows: R3 growth cones. Scale bars: 5 μm for FasII, GFP and RFP images; 1 μm for 24B10 images.
(TIF)

S3 Fig Variation of the standardized coordinate system across specimens. **(A)** Schematic of the standardized coordinate system. **(B)** Raw heel (filled circle) and target (hollow circle) grids for each bundle in three different specimens (taken from wild-type at 26hrs APF) aligned so that the “C” point (center of the standardized coordinate) is at (0,0) and T4 is on the X-axis. **(C)** Raw data for specimens 1–3 in (B) are transformed so that |C-T4| = 1 and ∡(T3,C,T4) = ∡(T4,C,T3’) = 1. Only data relevant to R3 and R4 are shown. **(D)** Centroids for specimens 1–3 in (C) are shown. Data used to generate this figure can be found in S1 Data.
(TIF)

S4 Fig Variation of the standardized coordinate system across time points. **(A)** Centroids of aligned raw heel and target positions of all bundles at given time points. Alignment is the same as S3A Fig. **(B)** Alignment of all raw centroids across time points. Increasing circle size indicates progression in time. **(C)** Schematic of the standardized coordinate system. **(D)** Polar plot of centroids of standardized coordinates across time points. Increasing circle size indicates progression in time. Data used to generate this figure can be found in S1 Data.
(TIF)

S5 Fig Extension phenotype of *sev>Fz* and *sev>N*^*ic*^ flies over time. **(A-B)** Representative images of wild-type-like (ctrl.) and fate-transformed (pert.) bundles in *sev>Fz* and s*ev>N*^*ic*^ flies at (A) 24 or (B) 28 hrs APF. Left four panes are confocal images of representative bundles. Right two panels are density maps of GFP (R4 cells) and RFP channel (R3 or R4 cells) after coordinate transformation. Image channels, intensity normalization, annotation and scale bars are as in S2 Fig.
(TIF)

S6 FigRepulsion model for determining growth cone extension angle.**(A)** Schematics of repulsion model. For R3, $\overset{⃑}{v_{1}}$ and $\overset{⃑}{v_{2}}$ represent repulsive forces from R2 and R4, respectively. For R4, $\overset{⃑}{v_{1}}$ and $\overset{⃑}{v_{2}}$ represent repulsive forces from R5 and R3, respectively. $\overset{⃑}{v_{p}}$: extension direction predicted from simulation; $\overset{⃑}{v_{m}}$: extension direction measured. **(B)** Difference between predicted and measured extension directions for data from 22, 24 or 26 hrs APF. $\overset{⃑}{v_{p}}=\alpha \;\overset{⃑}{v_{1}}+\beta \;\overset{⃑}{v_{2}}$ is used to calculate predicted extension directions. For the equal repulsion model, α = β = 0.5. For the weighted repulsion model, linear regression is performed to get α and β that best fit pooled data from wild-type measurements between 22 to 26 hrs APF. R3 regression result: α = 1.04, β = 0.44, R^2^ = 0.78; R4 regression result: α = 0.99, β = 0.65, R^2^ = 0.90. Data used to generate this figure can be found in S1 Data.(TIF)

S1 Table P-values for data used to create Figs 3 and 4. For Figs 3A and 4, significance is calculated using the two-sided Mann-Whitney test with p values adjusted by Holm-Bonferroni method. For Fig 3B, significance is calculated using the two-sided Mann-Whitney test. “R3/R4” stands for bundles with wild-type configuration; “R3/R3” and “R4/R4” stands for fate-transformed bundles that have two R3s or two R4s, respectively. “3” and “4” following “R3/R4”, “R3/R3” or “R4/R4” indicate the position of the growth cone.
(XLSX)

S1 Data Excel spreadsheet with numerical raw data underlying Figs 3, 4, S3, S4 and S6. (XLSX)
