# Supplementary figures and images for: Analysis of growth cone extension in standardized coordinates highlights self-organization rules during wiring of the Drosophila visual system
Source: PLoS Genet. 2021 Nov 3;17(11):e1009857. doi: 10.1371/journal.pgen.1009857 (PMC8565740; doi:10.1371/journal.pgen.1009857)

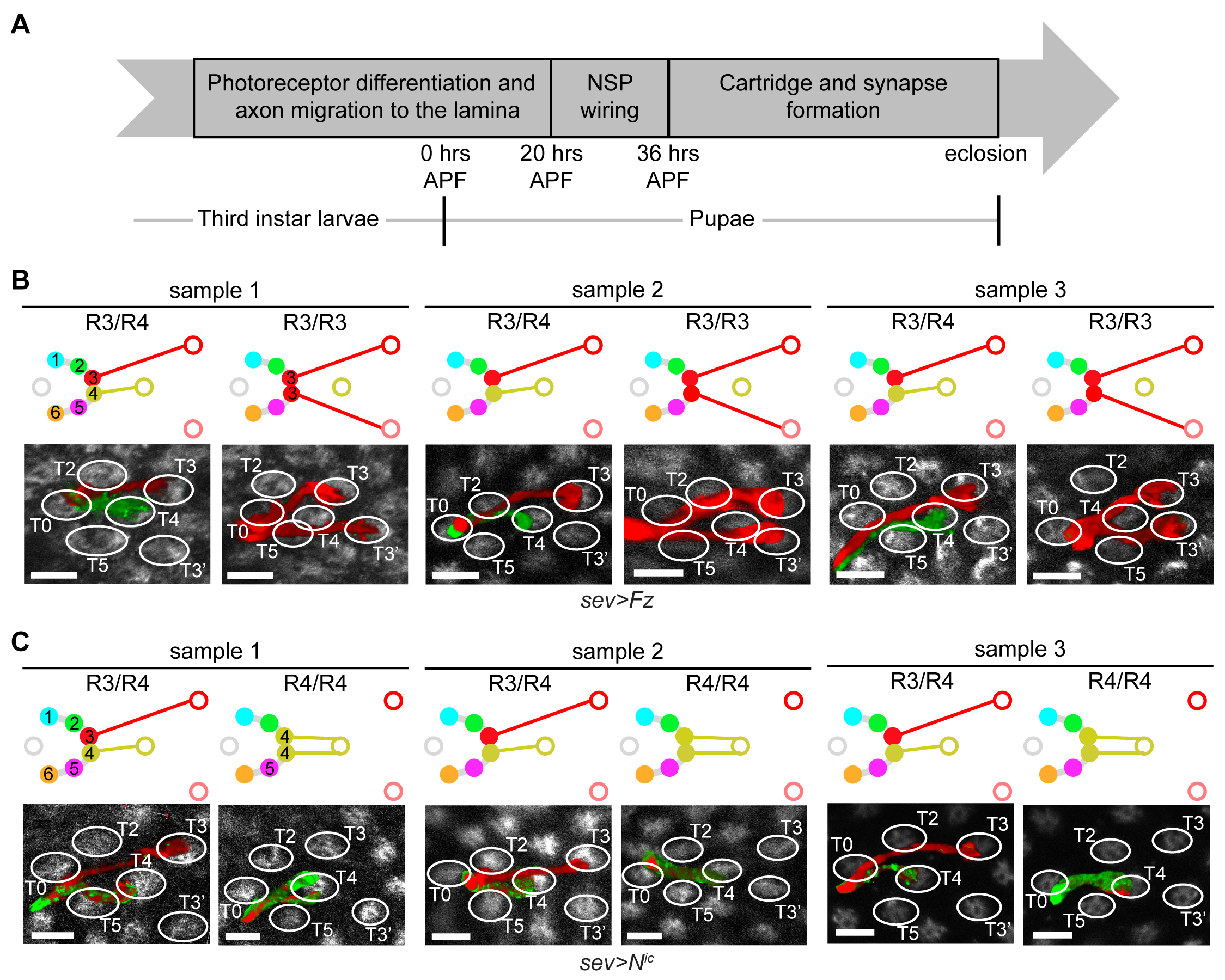

Supplement: S1 Fig — (A) Schematic for timing of NSP wiring. APF: “After Puparium Formation”. (B-C) Schematics (top panels) and confocal images (bottom panels) of bundles from three (B) sev>Fz (38 hrs APF) and (C) sev>Nic (45 hrs APF) specimens. Top panels: schematics of wild-type or altered wiring topology. Solid or open circles: starting points (‘heels”) or targets (respectively); colors coordinated between R cells and targets. T3’: target of fate-transformed R3s; T0: target located within the bundle of interest (though targeted by R cells from other bundles in NSP wiring). Bottom panels: confocal images of representative bundles. Photoreceptor growth cones are segmented and pseudo-colored (Materials and Methods) and intensity scaled for visualization. Red: sev>RFP expression; green: mδ0.5-GFP expression; white: Fasciclin 2 (FasII) antibody staining. White ellipses: targets. Scale bar: 5 μm. (TIF) [file pgen.1009857.s001.tif]

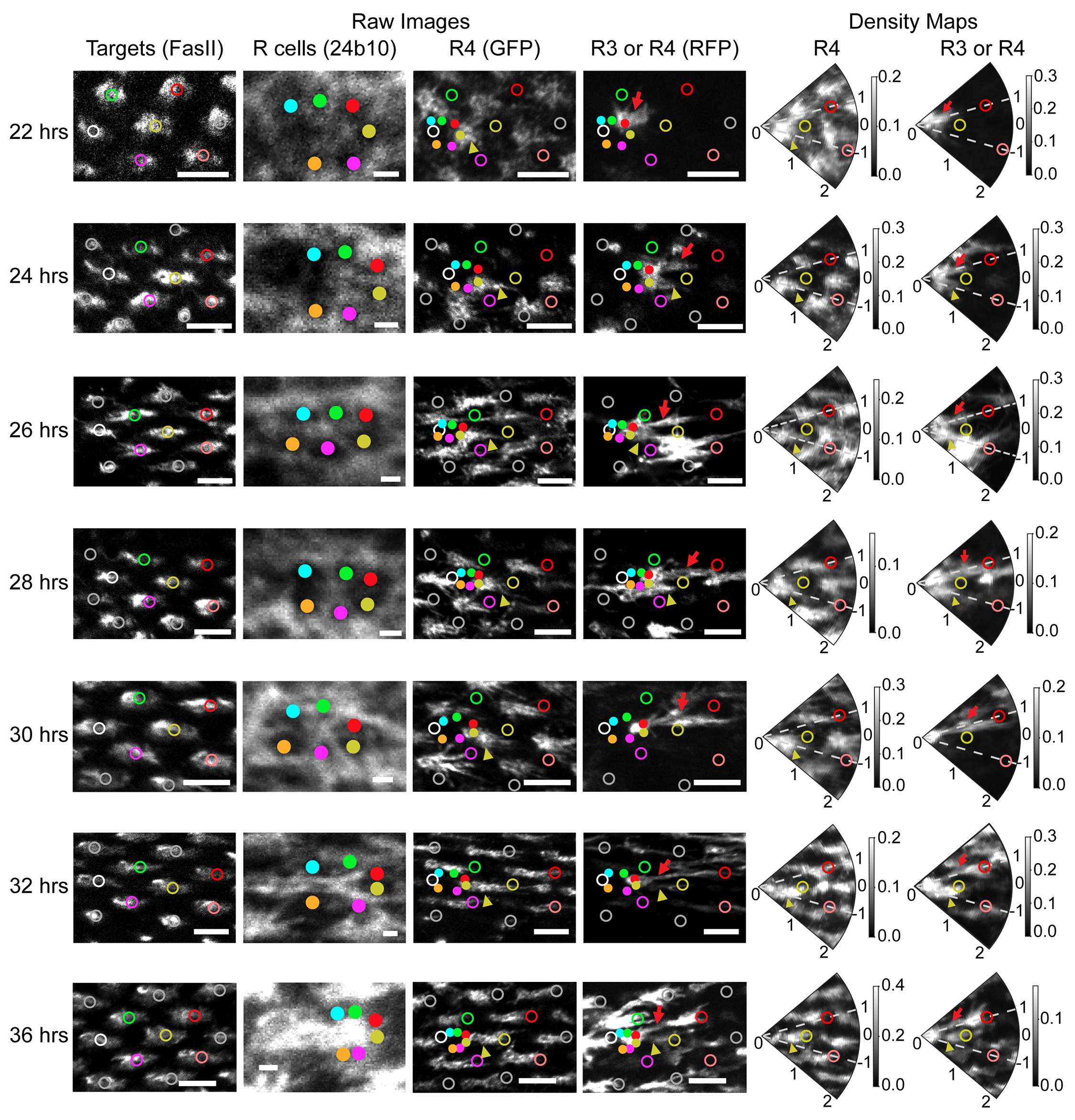

Supplement: S2 Fig — Representative images of bundles from wild-type flies from 22 to 36 hrs APF. Left: Raw images of representative bundles. From left to right: FasII channel labeling the target cells; 24B10 channel labeling membrane of all R cells; GFP channel labeling membrane of R4 cells; RFP channel labeling membrane of R3 or R4 cells. Right: Density maps of GFP (R4 cells) and RFP channel (R3 or R4 cells) after coordinate transformation. For visualization, intensity is scaled differently for each channel and for each sample. R-cells and targets are indicated and colored as in S1B Fig; white circles: T0; gray circles: other targets. Yellow arrowheads: R4 growth cones; red arrows: R3 growth cones. Scale bars: 5 μm for FasII, GFP and RFP images; 1 μm for 24B10 images. (TIF) [file pgen.1009857.s002.tif]

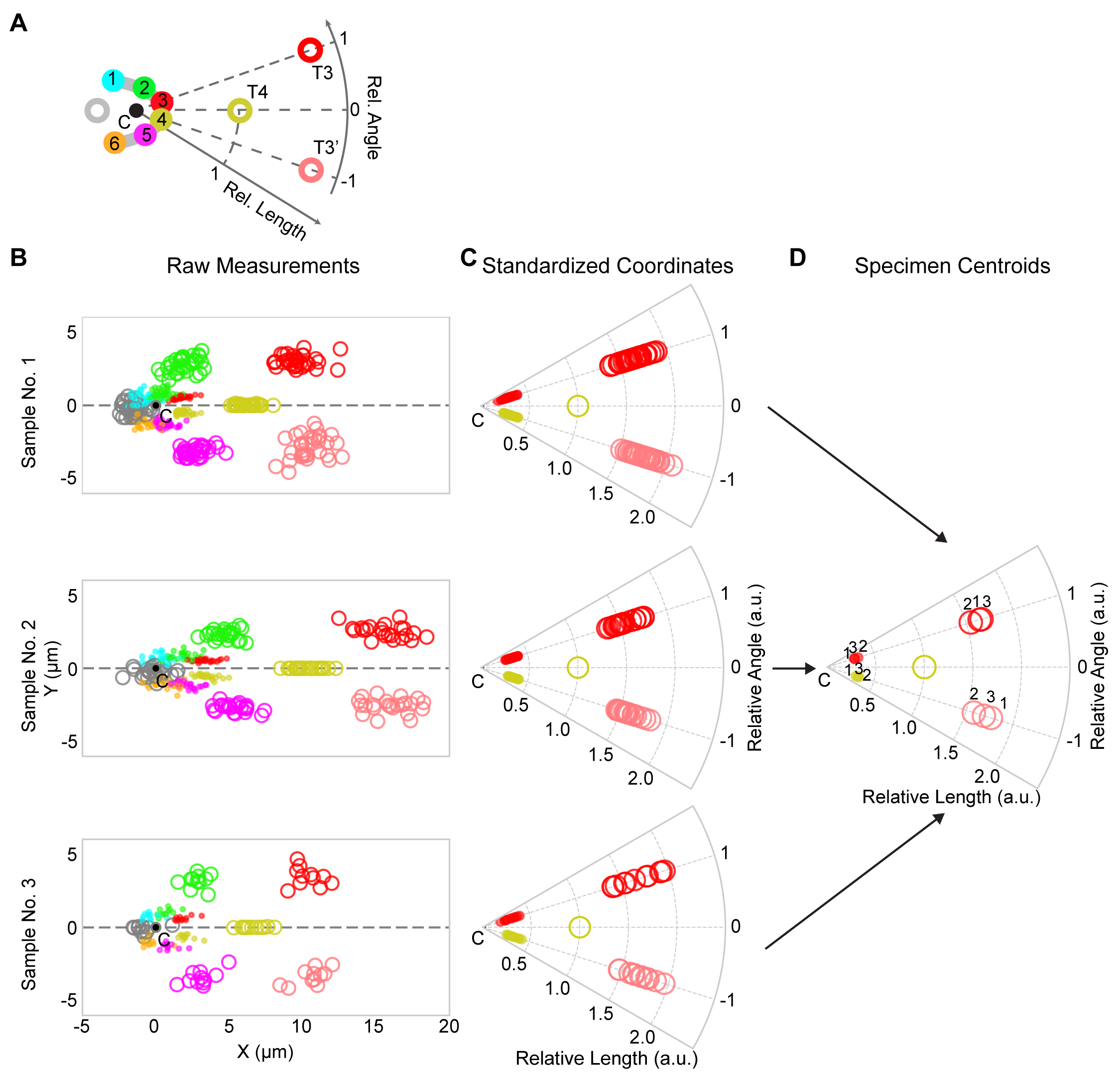

Supplement: S3 Fig — (A) Schematic of the standardized coordinate system. (B) Raw heel (filled circle) and target (hollow circle) grids for each bundle in three different specimens (taken from wild-type at 26hrs APF) aligned so that the “C” point (center of the standardized coordinate) is at (0,0) and T4 is on the X-axis. (C) Raw data for specimens 1–3 in (B) are transformed so that |C-T4| = 1 and ∡(T3,C,T4) = ∡(T4,C,T3’) = 1. Only data relevant to R3 and R4 are shown. (D) Centroids for specimens 1–3 in (C) are shown. Data used to generate this figure can be found in S1 Data. (TIF) [file pgen.1009857.s003.tif]

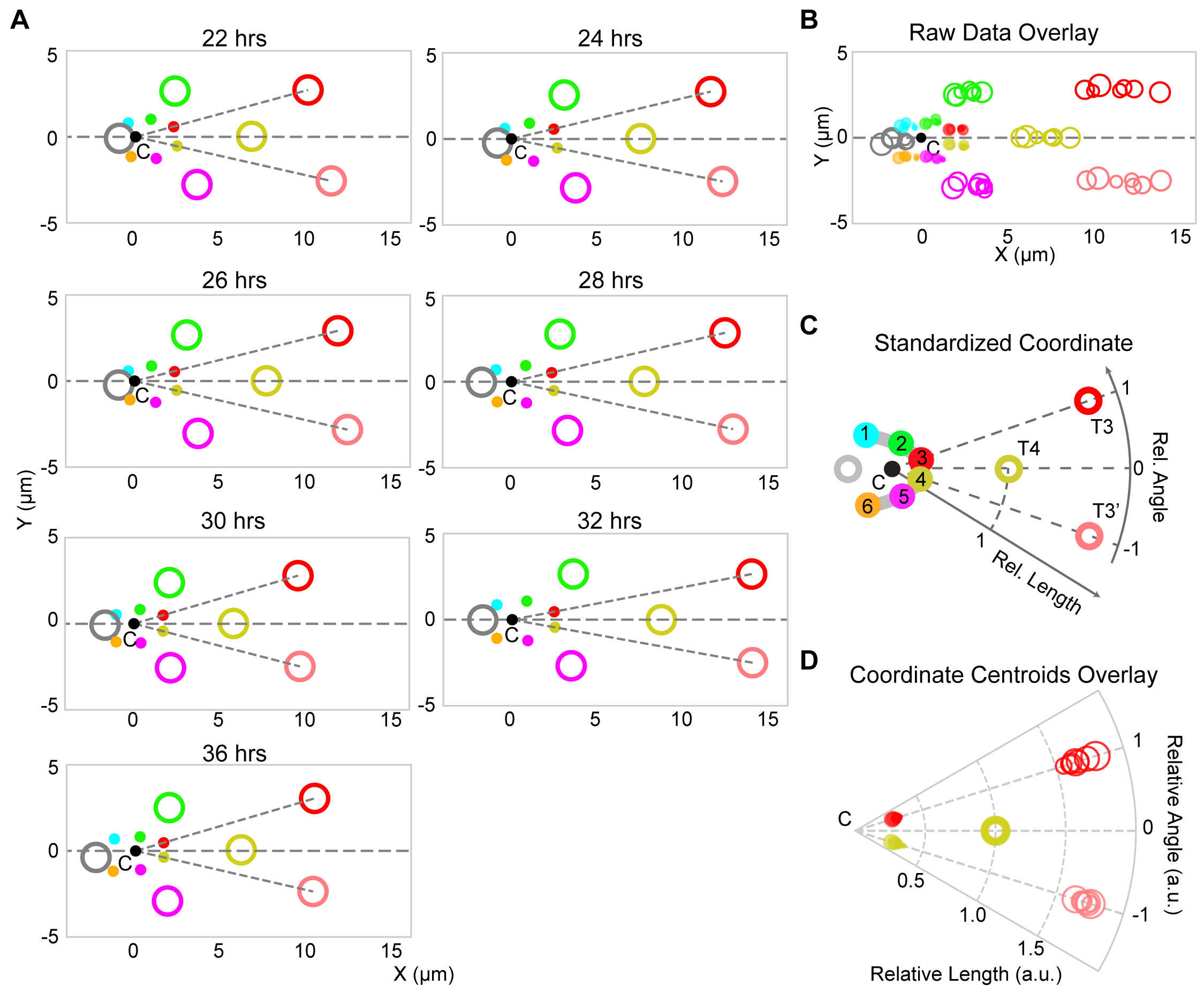

Supplement: S4 Fig — (A) Centroids of aligned raw heel and target positions of all bundles at given time points. Alignment is the same as S3A Fig. (B) Alignment of all raw centroids across time points. Increasing circle size indicates progression in time. (C) Schematic of the standardized coordinate system. (D) Polar plot of centroids of standardized coordinates across time points. Increasing circle size indicates progression in time. Data used to generate this figure can be found in S1 Data. (TIF) [file pgen.1009857.s004.tif]

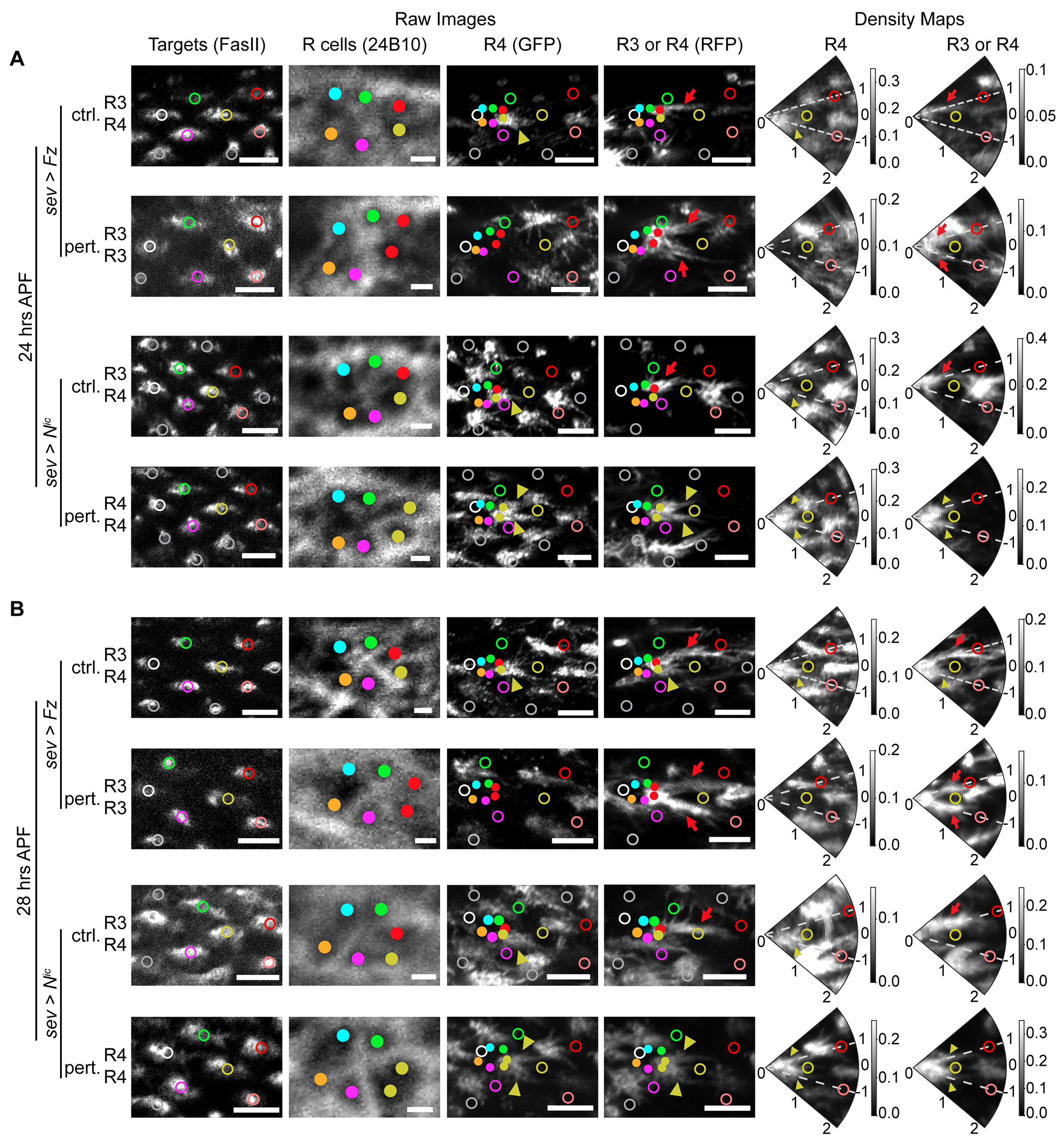

Supplement: S5 Fig — (A-B) Representative images of wild-type-like (ctrl.) and fate-transformed (pert.) bundles in sev>Fz and sev>Nic flies at (A) 24 or (B) 28 hrs APF. Left four panes are confocal images of representative bundles. Right two panels are density maps of GFP (R4 cells) and RFP channel (R3 or R4 cells) after coordinate transformation. Image channels, intensity normalization, annotation and scale bars are as in S2 Fig. (TIF) [file pgen.1009857.s005.tif]

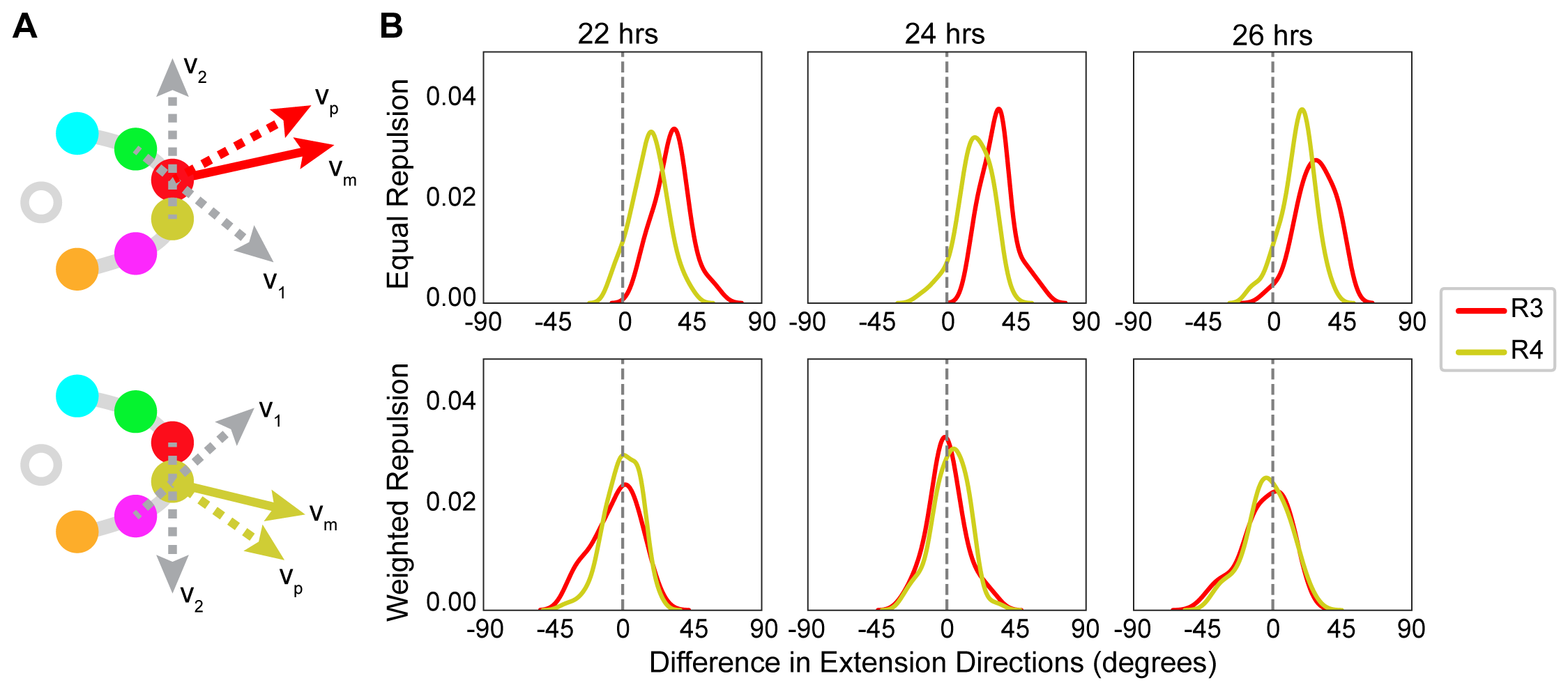

Supplement: S6 Fig — (A) Schematics of repulsion model. For R3, v1⃑ and v2⃑ represent repulsive forces from R2 and R4, respectively. For R4, v1⃑ and v2⃑ represent repulsive forces from R5 and R3, respectively. vp⃑: extension direction predicted from simulation; vm⃑: extension direction measured. (B) Difference between predicted and measured extension directions for data from 22, 24 or 26 hrs APF. vp⃑=αv1⃑+βv2⃑ is used to calculate predicted extension directions. For the equal repulsion model, α = β = 0.5. For the weighted repulsion model, linear regression is performed to get α and β that best fit pooled data from wild-type measurements between 22 to 26 hrs APF. R3 regression result: α = 1.04, β = 0.44, R2 = 0.78; R4 regression result: α = 0.99, β = 0.65, R2 = 0.90. Data used to generate this figure can be found in S1 Data. (TIF) [file pgen.1009857.s006.tif]
